# Peritoneal Dialysis-Related Peritonitis Caused by *Lysinibacillus sphaericus*

**DOI:** 10.1155/2024/2478832

**Published:** 2024-01-24

**Authors:** Teerawat Thanachayanont, Pailin Mahaparn, Tanyarat Teerapornlertratt, Teerachai Chantarojanasiri, Kriang Tungsanga

**Affiliations:** ^1^Bhumirajanagarindra Kidney Institute, Bangkok, Thailand; ^2^Department of Medicine, King Chulalongkorn Memorial Hospital, Chulalongkorn University, Bangkok, Thailand

## Abstract

Peritonitis is the major complication of peritoneal dialysis (PD) patients. *Staphylococcus* is the leading causative organism of PD-related peritonitis. However, there were more reports of unusual organisms causing peritonitis. Clinical features, management, and outcome of peritonitis from unusual organisms are important information. We reported herein a 72-year-old female patient who presented with fever, abdominal pain, and cloudy dialysate for 3 days. Upon admission, ceftazidime and vancomycin were given intraperitoneally. A preliminary report of blood and PD fluid culture showed the presence of Gram-positive bacilli. Her clinical status improved 48 hours after the commencement of the antibiotics. Subsequently, culture reports of blood and PD fluid showed *Lysinibacillus sphaericus* which was susceptible to vancomycin at a minimal inhibitory concentration of less than 0.25 *μ*g/mL. The patient was given intraperitoneal vancomycin for a total of 14 days. Then, the PD effluent was clear, and its cell count was below 100 cells/mm^3^ in 3 days. The patient did not have a recurrence of peritonitis after antibiotic discontinuation. The possibility of this organism infection is environmental contamination related to the patient's gardening activities.

## 1. Introduction

Peritonitis is an important complication and a leading cause of technical failure in peritoneal dialysis (PD) patients [1]. Common causative organisms are mostly bacteria and fungi. Among bacterial causes, *Staphylococcus* spp. and Gram-negative bacilli are frequently found [2]. However, peritonitis caused by unusual organisms has been increasingly reported [3, 4]. These are for instance *Pantoea agglomerans*, *Rhodotorula* spp., and *Morexella* spp. Management of these unusual organisms is mainly dependent on published literature [5]. *Lysinibacillus sphaericus*, formerly classified as *Bacillus* spp., is an aerobic, Gram-positive, rod-shaped, spore-forming bacterium that lives primarily in soil. *Lysinibacillus* spp. were initially considered as environmental contaminants and biocontrolling agent due to their insecticidal and larvicidal properties [6]. Yet, there were only 2 case reports of *L. sphaericus* infection in end-stage kidney disease (ESKD) patients, one due to hemodialysis catheter exit site infection [7] and the other from PD-associated peritonitis [8]. We reported herein a case of PD-related peritonitis secondary to *L. sphaericus* infection.

## 2. Case Report/Case Presentation

A 72-year-old woman had ESKD from diabetic kidney disease (DKD) and had been under PD treatment for 3 years with CAPD modality, 4 exchanges per day. She still had about 500–700 ml of urine per day. She received insulin therapy 24 units daily, with HbA1c ranging from 5.9 to 6.15%.

She did not take any immunosuppressive medication. She had not experienced any PD-related peritonitis (PDRP) or PD exit site infection. She presented with a history of fever, malaise, anorexia, abdominal pain, and cloudy dialysate for 3 days. There was no history of touch contamination or breaking aseptic technique. She did not have any pets in the household. Her leisure was gardening (watering and planting flowers). Upon admission, the body temperature was 38°C, heart rate 106 beats per min, respiratory rate 24 per min, and blood pressure 116/66 mmHg. She was well-oriented and mildly pale, with normal lungs and heart sounds. She had generalized abdominal tenderness and rebound tenderness. There was no purulent discharge from the PD catheter exit site.

**Figure 1:**
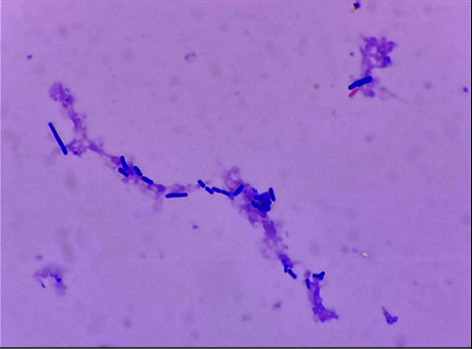
Gram stain finding of blood culture showing Gram-positive bacilli.

A complete blood count revealed a hemoglobin level of 10.8 g/dl, a white blood cell count of 13,400/mm^3^ with neutrophil 91%, and a platelet of 251,000/mm^3^. Her blood urea nitrogen (BUN) level was 33 mg/dl, serum creatinine (Cr) was 5.18 mg/dl, and serum albumin was 2.8 g/dL. The electrolyte panel revealed the serum sodium 128 mEq/L, serum potassium 3.2 mEq/L, and serum bicarbonate 24 mEq/L. The white blood cell (WBC) count of PD effluent was 1400/mm^3^ with 98% neutrophil. The Gram stain of PD fluid was negative. Two blood culture specimens were performed using an automated blood culture system (BACTEC). Peritoneal dialysis effluent was also sent for culture using the technique recommended by ISPD guidelines [5]. Upon admission, empirical antibiotics treatment with intraperitoneal ceftazidime 1.5 g and vancomycin 1 g (about 20 mg/kg) were given. Abdominal pain and febrile illness subsided within 24 hours. Thereafter, on the third hospitalization day, the white blood cell count of peripheral blood and PD effluent decreased to 7,300 and 40 cells/mm^3^, respectively. The serum sodium and potassium increased to 132 and 3.6 mEq/L, respectively. Gram-positive bacilli were detected in blood culture and peritoneal dialysis fluid specimens after 24 hours of inoculation (Gram stain of organism is shown in Figure 1), and these were subsequently identified as *Lysinibacillus sphaericus* after 96 hours of inoculation.

Antimicrobial susceptibility is shown in Table 1.

Due to clinical sepsis, intravenous vancomycin was given 1 dose on day 4 of admission which was about 96 hours after 1st dose of intraperitoneal vancomycin. Blood and PD fluid cultures were repeated on day 4 of admission and were negative for the organism. The patient was discharged from the hospital 5 days after admission. She and her caregiver were reemphasized on stringent hand hygiene techniques, particularly after gardening activities. During follow-up eight days after the infection, she was afebrile and asymptomatic. The PD effluent was negative for white blood cells. The plasma vancomycin level was 21.6 *μ*g/mL (about 5 days after the last dose of vancomycin). The antibiotic was changed to intraperitoneal vancomycin 1 g every 5 days to complete a total of 14 days of the antibiotic course. The patient was in good condition without any symptoms of peritonitis or fever until 60 days after the completion of the antibiotic.

## 3. Discussion/Conclusion

Although there are several uncommon bacterial organisms that can cause PD-related peritonitis, *L. sphaericus* has been rarely identified. To the best of our knowledge, this case is the second case report of PD-related peritonitis (PDRP) and the third case report of infection in end-stage kidney disease (ESKD) patients. *L. sphaericus* is reported to cause infection in immunocompromised hosts such as postsplenectomy, malignancy, or receiving bone marrow transplant patients [9–11]. *L. sphaericus* is an uncommon organism causing peritonitis in PD patients. Theoretically, ESKD patient is associated with immune dysfunction in many aspects [12]. In addition, the patient's diabetic condition is also a predisposing factor for infection. This is probably the reason why *L. sphaericus* can be pathogenic in these peritoneal dialysis patients. The clinical features of our case were typical of septicemic syndrome and were similar to those reported in previous publications [8]. Microbiological studies confirmed that *L. sphaericus* was causative organisms. Since *L. sphaericus* is commonly found in soil, it is likely that contamination from soil contact during or after her gardening activities should be the source of prime predisposing factor to this infection.

Besides improper hand hygiene after gardening that could have been a contributing factor to the infection, hypokalemia might also be an additional risk factor for peritonitis in our case. Hypokalemia among PD patients could pose a higher risk of peritonitis than those with normokalemia [13]. A previous systematic review a few years ago concluded that there was no convincing clinical study to show an association of hypokalemia with peritonitis in PD patients [14]. However, in multinational peritoneal dialysis outcomes and practice pattern study (PDOPPS), the largest observational prospective study on patients treated with PD, hypokalemia was more prevalent in Thailand. It was associated with protein-energy malnutrition, lower blood pressure, higher dialysis dose, prescription of high dose of diuretic, and lack of use of renin-angiotensin system inhibitor. Moreover, the rate of peritonitis is higher among PD patients with persistent hypokalemia [15]. Pichitporn et al. reported in a randomized controlled trial that oral potassium supplementation to Thai PD patients could significantly increase the median time to first peritonitis [16]. The latter was one of the first to demonstrate that correction of hypokalemia can lead to a lesser risk of peritonitis. Though the mechanistic pathway of hypokalemia in the development of peritonitis among PD patients has not been clearly elucidated, the low serum potassium identified in this case could have contributed to peritonitis caused by a low-virulence organism found in this case.

The patient was treated with two empirical antibiotics as suggested by ISPD guidelines. Intraperitoneal administration is the preferred route unless the patient is in sepsis condition [5]. Our patient initially presented without obvious clinical sepsis; thus, empirical antibiotics were given intraperitoneally. Even though there is no recommendation of the preferred route of antibiotic administration in patients who have concomitant bacteremia, systemic vancomycin was given to maintain the therapeutic level of vancomycin. The patient responded to management appropriately. Fever and abdominal pain were resolved within 48 hours. PD effluent leukocyte analysis showed improvement significantly by a decrement of more than 50% in 72 hours. These clinical and biological features were consistent with the nature of well-responded PDRP mentioned in the ISPD guideline [5]. Similar to the previous case report, our case response to antibiotic treatment is well and did not develop sepsis or septic shock despite concurrent bacteremia.

## 4. Conclusions

*L. sphaericus* is a low-virulence bacteria that can cause serious infection in PD patients. However, this infection responded to appropriate antibiotics well without relapse or recurrence features. A potential route of infection is environmental exposure. Thus, proper hand hygiene behavior should be strictly advised after the activities at risk for environmental exposures.

## Figures and Tables

**Table 1:** Antimicrobial susceptibility of *Lysinibacillus sphaericus* in our case.

Antibiotics	Susceptibility	MIC (*μ*g/mL)
Gentamicin	S	<2
Ampicillin	S	<0.12
Trimethoprim-sulfamethoxazole	S	<0.5
Ciprofloxacin	S	<0.5
Levofloxacin	S	<0.25
Penicillin	S	<0.06
Tetracycline	S	<2
Erythromycin	S	<0.25
Clindamycin	S	<0.5
Vancomycin	S	<0.25
